# A versatile “all-in-one” episomal system simplifies genome engineering in diatoms

**DOI:** 10.1093/plphys/kiag386

**Published:** 2026-06-17

**Authors:** Mireia Uranga

**Affiliations:** Assistant Features Editor, Plant Physiology, American Society of Plant Biologists, Rockville, MD 20855-2768, United States; Institute for Integrative Systems Biology (I2SysBio), Universitat de València-CSIC, 46980 Paterna, Valencia, Spain

Oceans, rivers, and lakes on our planet are covered by an “aquatic” rainforest invisible to the naked eye: diatoms. This diverse group of photosynthetic microalgae accounts for only 0.1% of total plant biomass but contributes over 20% of global net primary production and is a key driver of biochemical cycles (Stiller et al 2014; Falciatore et al 2020). Besides their ecological relevance, diatoms produce silica structures and high-value metabolites like omega-3 fatty acids and carotenoids, offering significant potential for biotechnological applications.

The diatom *Thallassiosira pseudonana* was the first to have its genome sequenced and has since become a model system for researchers (Armbrust et al 2004). Lately, a variety of molecular genetic tools have been developed to unravel key aspects of diatom cell biology, metabolism, and adaptability to environmental changes. In addition to well-established methodologies for genetic transformation, a variety of expression vectors are now available for fluorescent protein (FP) tagging and CRISPR-Cas9 genome editing in diatoms (Li et al 2025). However, because current tools lack an integrated framework, multiple vectors need to be delivered for specific tasks, which limits opportunities for genetic engineering in diatoms.

Recently in *Plant Physiology*, Nam et al (2026) expanded the existing molecular resources for diatoms by developing a single-vector episomal toolkit that supports simultaneous FP tagging and genome editing in *T. pseudonana*. The system uses Golden Gate modular cloning to hierarchically assemble multiple DNA fragments in a standard expression vector that contains 3 core elements: (i) the episomal maintenance cassette (*CEN6-ARSH4-HIS3*) enables self-replication and avoids random integration into the host genome, (ii) the origin of Transfer (*oriT*) mediates vector transfer during conjugation, and (iii) the N-acetyl transferase (*NAT*) gene provides resistance to the antibiotic nourseothricin. To be compatible with previously developed systems, the expression vector follows the Plant/*Chlamydomonas* MoClo syntax (Patron et al 2015; Crozet et al 2018): the episomal components *CEN6-ARSH4-HIS3* and *oriT* are combined in position 1, the *NAT* marker is inserted in position 2, and positions 3 to 5 are occupied with diverse combinations of transcriptional units to support a broad range of applications (Fig. 1). The assembled episome is transformed into *Escherichia coli* cells containing a mobility plasmid before its transfer to *T. pseudonana* by conjugation.

**Figure 1:**
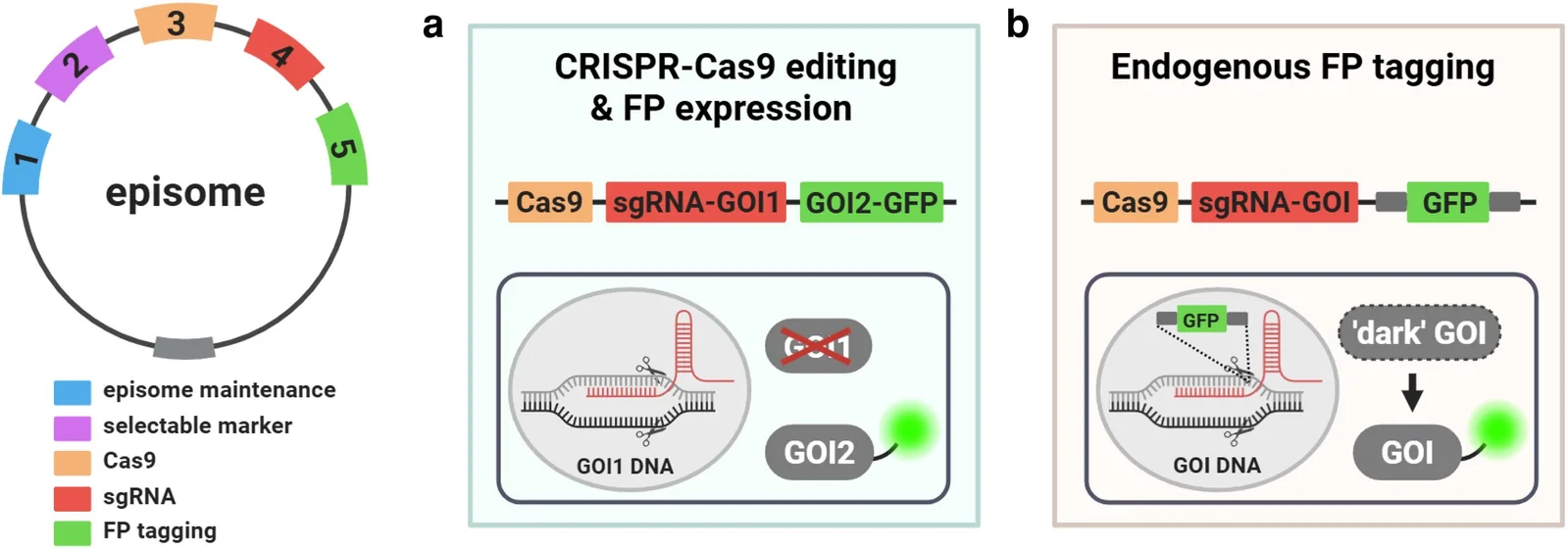
A single-vector episomal framework for multipurpose genome engineering in diatoms. Following Golden Gate modular cloning, multiple DNA fragments are hierarchically assembled into an episome, which is delivered into *T. pseudonana* via conjugation. Positions 1 and 2 are essential for episome maintenance and transformant selection, while positions 3 to 5 can be customized with different transcriptional units for a variety of applications, including a) simultaneous gene knockout and FP expression and b) scarless endogenous FP tagging via homology-directed repair knock-in. “Dark GOI” refers to the native, untagged protein present in the cell. GOI, gene of interest. Adapted from Fig. 1, Nam et al. (2026), and created in BioRender.

Initially, the authors validated their toolkit for endogenous FP tagging by expressing an episomal copy of Bestrophin-like protein 2 (BST2) C-terminally fused to GFP. In the green alga *Chlamydomonas reinhardtii*, BSTs are located in pyrenoid-associated thylakoids and mediate HCO_3_^-^ transport into the lumen, where a carbonic anhydrase provides CO_2_ directly to Rubisco for its fixation (Mukherjee et al 2019). After conjugation and selection of *T. pseudonana* transformants, confocal imaging showed that BST2-GFP adopted an elongated structure at the center of the chloroplast. This observation supports that diatom BSTs might supply inorganic carbon in a similar way to reports in other algae. Furthermore, the authors confirmed that episome-encoded dual-color protein tagging is possible by fusing the pyrenoid-enriched protein diatom pyrenoid component 1 (DPC1) and Rubisco small subunit (rbcS) to GFP and mScarlet-I, respectively.

Subsequently, Nam et al (2026) aimed to perform simultaneous CRISPR-Cas9 editing and FP tagging using a bifunctional episome (Fig. 1a). For this, they assembled into the same vector a CRISPR-Cas cassette targeting the *DPC1* locus and a different transcriptional unit expressing rbcS-GFP. Although sequencing analysis confirmed *DPC1* disruption, the localization of rbcS-GFP was not altered in the knockout mutants, contradicting the proposed structural role of DPC1 in pyrenoid assembly (Nam et al 2024). These results demonstrate that a single episomal vector can mediate gene editing and FP expression at once, offering a simplified workflow for genetic complementation and colocalization studies in *T. pseudonana*.

Although ectopic FP expression is widely employed for protein localization studies in diatoms, overexpressing the target gene through a strong constitutive promoter can alter protein structure and lead to nonspecific interactions with other proteins. Conversely, scarless FP insertion preserves the expression by native *cis* and *trans* regulatory elements (Bukhari and Müller 2019). Considering the absence of endogenous tagging systems in diatoms, Nam et al (2026) followed a CRISPR-Cas9-guided knock-in (KI) approach using the episomal toolkit (Fig. 1b). They designed a single guide RNA targeting 40 bp upstream of the BST2 stop codon and added homology arms flanking the cutting site. A successful knock-in would result in GFP insertion 13 amino acids before the BST2 stop codon, and the disruption of the target region would prevent recutting by Cas9. Initial screening of transformants by flow cytometry revealed up to 58% knock-in efficiency for *BST2*, and BST2-GFP^KI^ was detected as a linear structure at the center of the chloroplast, discarding an impact of GFP on protein localization. Since BST2 expression in the knock-in lines is under the control of native regulatory elements, the authors next explored whether it could be influenced by environmental factors. Interestingly, BST2-GFP^KI^ lines exhibited increased fluorescence when grown at low CO_2_ levels, in accordance with previous studies suggesting that BST2 expression and translation are induced under low carbon supply (Nigishi et al 2024). These findings suggest that fluorescence intensity could serve as an indicator of changes in protein abundance in response to environmental stimuli.

In summary, Nam et al (2026) developed a versatile, single-vector episomal toolkit that promotes genetic engineering applications in diatoms, including FP expression, gene editing, and scarless endogenous FP tagging. The system's high efficiency simplifies the generation of knock-in lines and paves the way for additional genetic modifications, such as replacements, rearrangements, and neofunctionalization of specific target sites. However, further efforts should address variations in knock-in efficiencies across the genome and alternative strategies beyond flow cytometry to enable absolute protein quantification. Tackling these aspects will establish endogenous tagging as a routine method for native-context cell biology research in diatoms, setting the benchmark for future genome-scale studies.

## Data Availability

None required.
